# Videos on YouTube, Bilibili, TikTok as sources of medical information on Hashimoto’s thyroiditis

**DOI:** 10.3389/fpubh.2025.1611087

**Published:** 2025-10-15

**Authors:** Shiqi Wang, Siyu Jia, Yanjun Su, Ruochuan Cheng

**Affiliations:** ^1^Department of Thyroid Surgery, Clinical Research Center for Thyroid Diseases of Yunnan Province, The First Affiliated Hospital of Kunming Medical University, Kunming, China; ^2^Hend and Neck Tumor Center, Hefei Cancer Hospital, Chinese Academy of Sciences, Hefei, Anhui, China

**Keywords:** Hashimoto’s thyroiditis, hypothyroidism, social media, health education, public health

## Abstract

**Introduction:**

Hashimoto’s thyroiditis (HT), a common autoimmune thyroid disorder, is widely discussed on video-sharing platforms. However, user-generated content about HT lacks systematic scientific validation. This study evaluates the reliability and quality of HT-related videos on three major social media platforms: YouTube, Bilibili, and TikTok.

**Methods:**

Between December 1, 10, 2024, the top 200 videos meeting the criteria retrieved under default search settings using a newly registered user account were included for each platform. These videos were from 107 YouTube accounts, 56 Bilibili accounts and 90 TikTok accounts. Metrics including video parameters and creator profiles were recorded. Content quality was evaluated using five validated assessment tools: PEMAT (Patient Education Materials Assessment Tool), VIQI (Video Information and Quality Index), GQS (Global Quality Score), mDISCERN (modified DISCERN), and JAMA (Journal of the American Medical Association) standards.

**Results:**

TikTok videos showed the highest audience engagement. YouTube had more team-based accounts (43.9%), while TikTok and Bilibili predominantly featured individual accounts, with TikTok featuring a notably higher proportion of verified individual accounts (86.7%). Solo narration was the most common video style across YouTube (62.5%) and TikTok (70.0%), while in Bilibili, it was the medical scenario. In contrast, YouTube and Bilibili offered a broader range of content, including TV programs, documentaries, and educational courses. The varying emphases of different assessment tools rendered it difficult to determine which platform boasts the highest content quality, but the video quality scores across all platforms are not satisfying. Additionally, we found that content produced by verified creators was of higher quality compared to that of unverified creators, with this trend being particularly evident among individual accounts.

**Conclusion:**

Social media platforms provide partial support for the dissemination of health information about HT, but the overall video quality remains suboptimal. We recommend that professional creators pursue platform certification to enhance the dissemination of high-quality HT-related videos.

## Introduction

Social media is an important way to share health information, especially through videos (1). A large number of studies evaluating the information related to thyroid diseases on social media have been indexed in PubMed, such as hypothyroidism (2) and hyperthyroidism (3), Grave’s disease (4, 5), and thyroid cancer (6–11).

Hashimoto’s thyroiditis (HT), alternatively referred to as chronic lymphocytic thyroiditis, autoimmune thyroiditis or Hashimoto’s disease, represents a thyroid-specific autoimmune disorder defined by three principal pathological features: thyroid gland enlargement, lymphocytic infiltration within thyroid parenchyma, and detectable serum antibodies targeting thyroid-specific antigens. Although the incidence of HT has decreased in recent years, it remains high (216.0 to 161.5 for 2000–02 to 2017–19; standardized incidence per 100,000 person-years). Across the full study period, the median age at diagnosis for HT was 58 years (IQR 43–75), indicating an age distribution weighted toward older individuals (12). Another epidemiological survey covering 31 major cities in China shows that 10.19% of the total population has positive TPOAb (anti-thyroid peroxidase antibody), and 9.7% has positive TgAb (anti-thyroglobulin antibody) (13), both of which are markers of autoimmune thyroid disease. As the most prevalent autoimmune thyroid disorder, HT represents the primary driver of hypothyroidism in regions with sufficient iodine intake (14, 15).

As HT is one of the common causes leading to hypothyroidism, the most relevant research to our study was conducted by Dulak et al. in 2023 (2). They assessed the quality of 96 hypothyroidism-related videos on YouTube using the DISCERN tool and Video Power Index (VPI) and concluded that the overall quality of YouTube videos regarding hypothyroidism was poor (2). However, this study focused on a single platform, comparative analyses across multiple platforms are still limited, and only one scale was used to evaluate the video quality.

This study offers the first comprehensive analysis of HT-related videos on three large video platforms, YouTube, Bilibili, and TikTok. We thoroughly examine key characteristics and evaluate the content quality of these videos to promote the development of public health education.

## Materials and methods

### Search strategy

**Figure 1:**
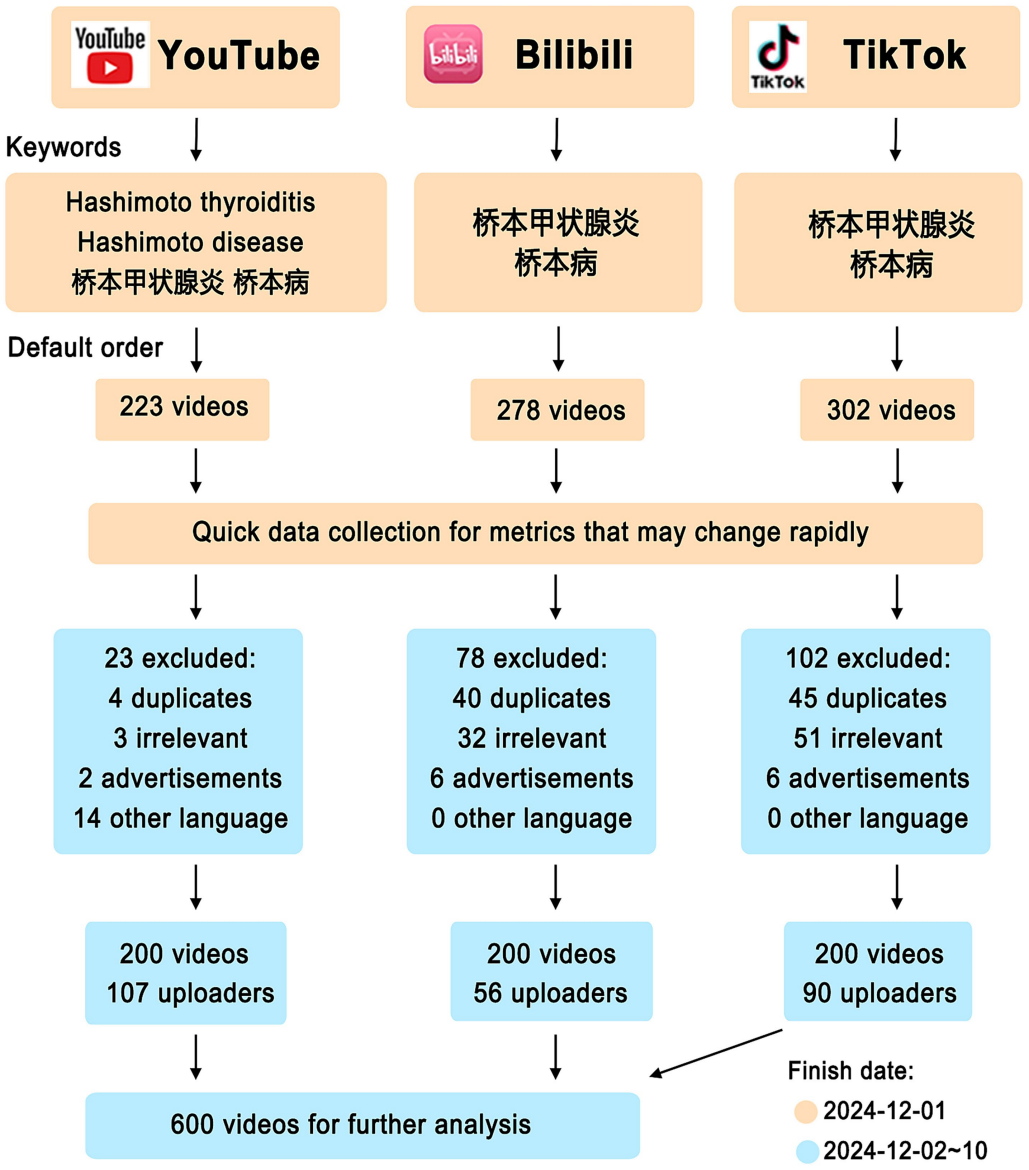
Search strategy for videos on Hashimoto’s thyroiditis.

Figure 1 outlines the search protocol. Briefly, we conducted video searches on three platforms on October 1, 2024. The English keywords are “Hashimoto’s thyroiditis” and “Hashimoto’s disease,” and the corresponding Chinese keywords are “桥本甲状腺炎” and “桥本病.” On YouTube, searches were performed using both English and Chinese keywords concurrently. In contrast, on Bilibili and TikTok, only Chinese keywords were utilized for the search process. To minimize algorithmic bias, we cleared the browser history and used a newly registered account. Videos were reviewed in the default order as determined by the platforms’ algorithms.

### Data collection

Data collection is divided into two phases. Phase one, lasting 1 day, focuses on capturing data that undergoes rapid temporal changes, such as views, likes, coins, collections, shares, and the number of the uploader’s followers. These metrics reflected the audience engagement of a video or an account. Phase two, spanning 9 days, involves screening out the videos according to the exclusion criteria, collecting time-invariant data (titles, release dates, video lengths, uploader IDs, certification status, uploaders’ type (individual or team), video topics, video styles), and assessing the video quality. Video styles were categorized into seven types based on the method proposed by Liu et al., including Solo narration, Animation, PPT presentation, etc. (16). Video topics were classified into five categories (e.g., Etiology/Prevention; Symptoms; Diagnosis; Treatment/Prognosis; Others), using a study-specific framework developed by the authors. Some videos covered more than one topic. Thus, the number of topics covered by each video was also analyzed. The one that took up the longest duration of the video was defined as the main topic. Detailed information on the methodology is listed in Supplementary file 1.

Two evaluators independently assessed and rated video quality using validated measurement tools. Discrepancies in ratings were adjudicated through consultation with a third researcher to establish consensus. These tools were: the Patient Education Materials Assessment Tool (PEMAT), the Video Information Quality Index (VIQI), the Global Quality Scale (GQS), the modified DISCERN instrument (mDISCERN), and the Journal of the American Medical Association (JAMA) benchmark criteria. The complete contents of the tools are documented in Supplementary files 2, 3. Video characteristics and uploader characteristics are the secondary outcomes. Video contents and quality assessment are the primary outcomes.

### Statistical analysis

Data were analyzed using IBM SPSS 25.0 and GraphPad Prism 8. Data were considered non-normally distributed when the null hypothesis of the Shapiro–Wilk test was rejected, and the remaining data were treated as approximately normal for practical purposes. Normally distributed data were expressed as mean±SD and non-normal data as median and 25th ~ 75th percentiles (M [P25 ~ P75]). The Mann–Whitney test compared non-parametric variables between two groups, and the Kruskal-Wallis Test for three groups with the Dunn Test for multiple comparisons. As this was an exploratory analysis with no pre-specified primary outcomes, multiple comparison corrections were not applied. A nominal *p*-value < 0.05 was considered to suggest a potential signal. Categorical variables were presented as counts and percentages (n (%)) and analyzed through the Chi-square test with the Bonferroni method for multiple comparisons. We used weighed *κ* and ICC (Intraclass Correlation Coefficient) to quantify the agreement between the two raters (more details see in Supplementary file 2). However, due to the limitations of this study as described in the discussion, a statistically significant difference might not be detected, but did not confirm the absence of a difference.

## Results

### Video characteristics

After applying the inclusion and exclusion criteria, the top 200 videos retrieved under default search settings using a newly registered user account were included for each platform, because users typically scroll through approximately 100 ~ 200 recommended videos for a given keyword (17, 18). Statistical testing confirmed that all continuous variables exhibited non-normal distributions (Shapiro–Wilk test, *p*-value < 0.05). YouTube videos demonstrated the longest durations. Due to the restrictions of each platform, some data were not available. TikTok achieved the highest levels of user engagement, as indicated by likes, comments, collections, and shares. Interestingly, despite YouTube having a higher amount of views, thumbs up and comments compared to Bilibili, the ratio of comments to views was lower than that of Bilibili (Table 1).

**Table 1:** Characteristics of videos related to Hashimoto’s disease on YouTube, Bilibili, and TikTok.

Platforms	YouTube (N_1_ = 200)	Bilibili (N_2_ = 200)	TikTok (N_3_ = 200)	*p*-value		
				P_Y-B_	P_B-T_	P_Y-T_
Video length (seconds)	243 [110 ~ 603]	152 [98 ~ 262]	80 [54 ~ 130]	**0.003**	**<0.001**	**<0.001**
Views	3,894 [158 ~ 32,769]	1754 [472 ~ 5,238]	**-**	**0.008**	**-**	**-**
Thumbs up	98 [0 ~ 714]^*^	22 [6 ~ 69]	495 [164 ~ 2014]	**<0.001**	**<0.001**	**<0.001**
Comments	5 [0 ~ 89]^**^	2 [0 ~ 13]	33 [12 ~ 180]	**0.009**	**<0.001**	**<0.001**
Collections	**-**	20 [6 ~ 70]	276 [55 ~ 1,042]	**-**	**<0.001**	**-**
Shares	**-**	11 [2 ~ 36]	154 [37 ~ 798]	**-**	**<0.001**	**-**
Coins	**-**	2 [0 ~ 8]	**-**	**-**	**-**	**-**
Thumbs-up / Views (%)	1.53 [0.00 ~ 3.33]^*^	1.43 [0.88 ~ 2.13]	**-**	0.643	**-**	**-**
Comments / Views (%)	0.06 [0.00 ~ 0.29]^**^	0.16 [0.00 ~ 0.37]	**-**	**0.024**	**-**	**-**
Collections / Views (%)	**-**	1.35 [0.93 ~ 2.12]	**-**	**-**	**-**	**-**
Shares / Views (%)	**-**	0.63 [0.27 ~ 1.08]	**-**	**-**	**-**	**-**
Coins / Views (%)	**-**	0.08 [0.00 ~ 0.26]	**-**	**-**	**-**	**-**

Data were presented as Median [P25 ~ P75]. Bold text means the *p*-value<0.05. “-” means “not available.”

Cells are marked pink when the value shows a nominally significant signal of being higher than others (*p* < 0.05) and yellow when showing a nominally significant signal of being lower (*p* < 0.05); no color is applied when no nominally significant signal is detected.

*p*-values between two groups were obtained from Mann–Whitney U test. *p*-values between three groups were obtained Kruskal-Wallis Test, and Dunn Test was used for multiple comparisons. P_Y-B_, YouTube versus Bilibili; P_B-T_, Bilibili versus TikTok; P_Y-T_, YouTube versus TikTok.

* Excluded: 7 videos on YouTube turned off the function of thumbs-up. **Excluded: 5 videos on YouTube turned off the function of comments.

### Uploader characteristics

**Figure 2:**
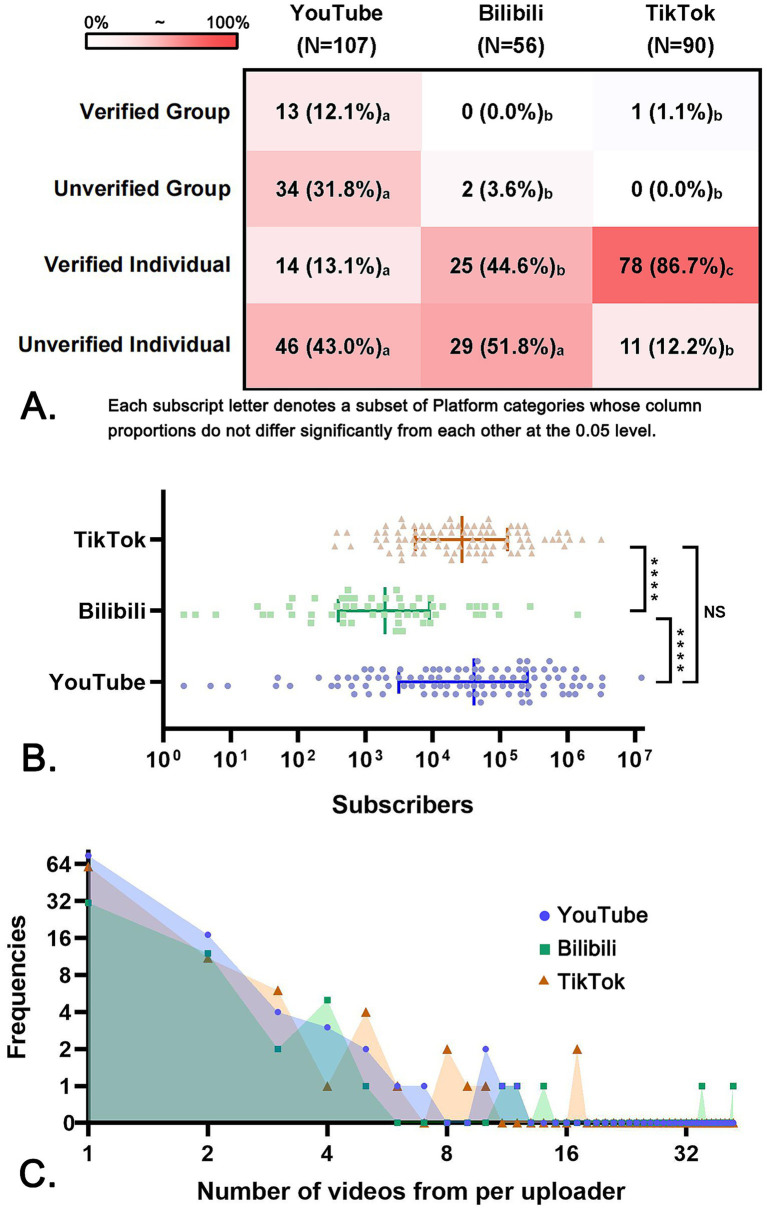
Uploader characteristics on YouTube, Bilibili, and TikTok. **(A)** Heat map: Distribution of uploader types per platform (*N* = number of accounts). **(B)** Scatter plot: Number of subscribers. The long vertical line represents the median, and the short vertical line represents the interquartile range. **(C)** The number of videos included in this study for per uploader. *p-value<0.05, **p-value<0.01, ***p-value<0.001, ****p-value<0.0001, NS, not significant.

The videos enrolled in this study were uploaded by 107 accounts on YouTube, 56 on Bilibili, and 90 on TikTok. Figure 2A shows the heat map of uploader categories across the three platforms. It’s interesting to note that 43.9% of YouTube uploaders were team accounts, while nearly most of the uploaders on Bilibili and TikTok were individuals. Notably, TikTok owned the largest ratio of verified individual creators, and all of them were doctors. Bilibili uploaders owned the smallest number of subscribers (1943 [395 ~ 8,920]), while YouTube (40,700 [3,120 ~ 255,000]) and TikTok (27,000 [5,472 ~ 127,750]) uploaders had more (Figure 2B). The frequency distribution diagram (Figure 2C) reveals that most uploaders had only one video included in our study. Nevertheless, a few uploaders demonstrated notably high productivity; the highest output was observed from a Bilibili uploader who released 42 videos enrolled in our study.

### Video content

**Figure 3:**
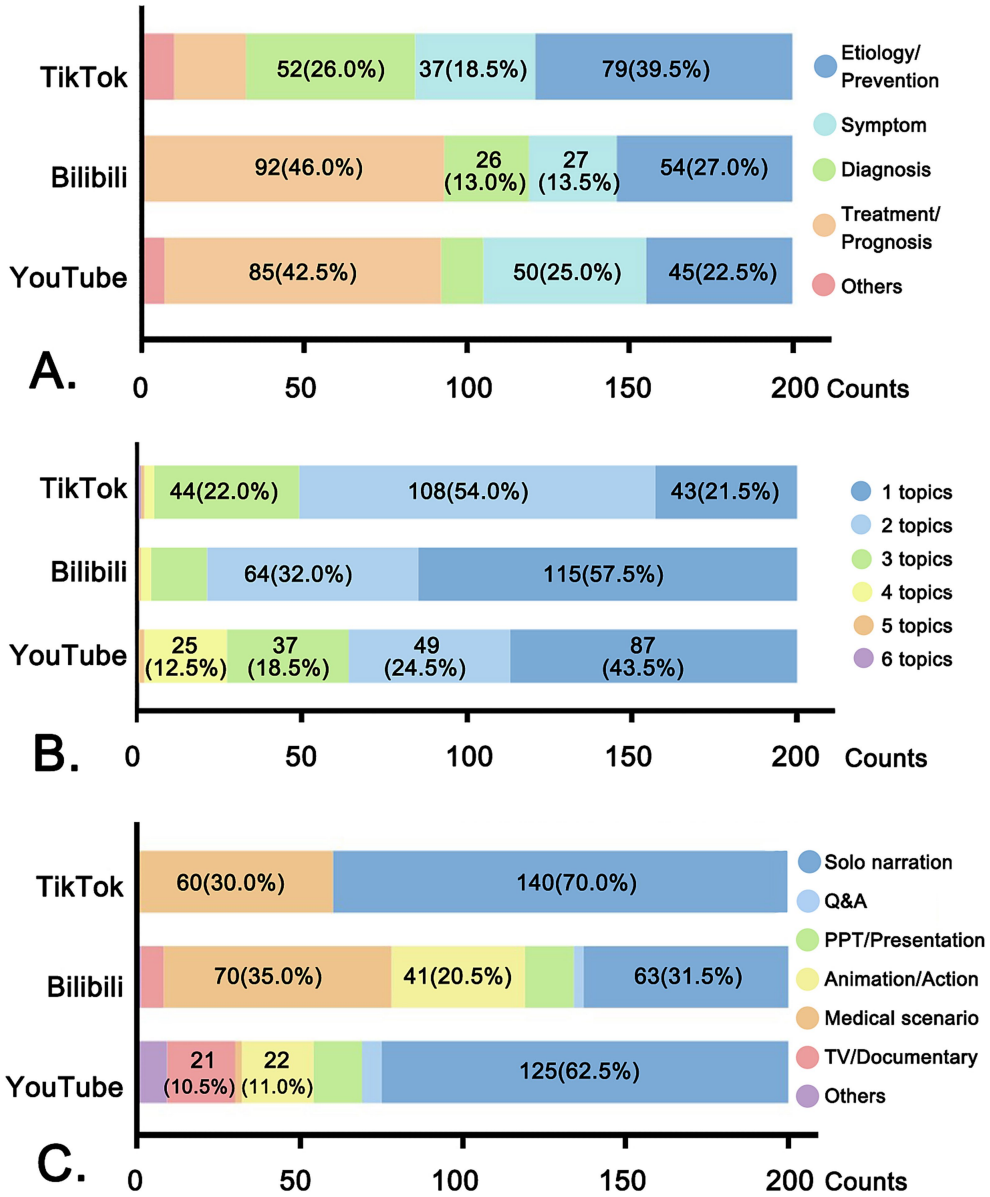
Content analysis of videos on YouTube, Bilibili, and TikTok. **(A)** Main topic types. **(B)** Number of topics covered in each video. **(C)** Video style types.

In general, the topic of treatment/prognosis was more popular on YouTube and Bilibili than on TikTok, where etiology/prevention dominated the main topics (Figure 3A). Most videos covered 1 ~ 2 topics, while 32% YouTube videos, 24.5% TikTok videos, and only 10.5% Bilibili videos covered more than three topics (Figure 3B).

As for video production styles, though the solo narration was the predominant format across all platforms, YouTube and Bilibili featured a greater variety of formats, including TV shows/documentaries, animations, and PPT/presentations (Figure 3C).

### Video quality

The agreement between the two evaluators was substantial (Supplementary file 2). The varying emphases of different assessment tools rendered it difficult to determine which platform boasts the highest content quality (Table 2).

**Table 2:** Quality assessment of videos related to Hashimoto’s disease on YouTube/ Bilibili/ TikTok.

Platforms	YouTube (*N* = 200)	Bilibili (*N* = 200)	TikTok (*N* = 200)	*p*-value
PEMAT	75 [69 ~ 77]_a_	73 [64 ~ 75]_b_	83 [77 ~ 85]_c_	**<0.001**
VIQI	11 [10 ~ 13]_a_	11 [10 ~ 11]_b_	11 [10 ~ 12]_b_	**<0.001**
GQS	3 [3 ~ 3]_a_	3 [3 ~ 4]_b_	3 [3 ~ 4]_c_	**<0.001**
mDISCERN	3 [3 ~ 3]_a_	3 [3 ~ 3]_b_	3 [3 ~ 3]_b_	**<0.001**
JAMA	1 [1 ~ 2]_a_	2 [2 ~ 2]_b_	2 [2 ~ 2]_b_	**<0.001**

Data were presented as Median [P25 ~ P75]. Bold text means the *p*-value<0.05.

*p*-values were obtained from Kruskal-Wallis Test and Dunn Test was used for multiple comparisons: Each subscript letter denotes a subset of “Platform” categories whose column proportions do not differ significantly from each other at the 0.05 level.

Pink indicates groups that are significantly higher than at least one other group (*p* < 0.05, Dunn test), and yellow indicates groups that are significantly lower than at least one other group (*p* < 0.05, Dunn test). If two cells share the same color, it means that both groups differ significantly from at least one other group, but they are not significantly different from each other.

However, we found that the video quality significantly varied across different types of uploaders (Table 3). There were 5.3% (32/600) videos with obviously unbalanced or misleading information, which were all from the unverified accounts. For instance, some videos claimed that a high-iodine diet can exacerbate Hashimoto’s disease without mentioning that these patients also need appropriate iodine. Some videos over-emphasized the connection between food allergies and Hashimoto’s disease, especially gluten-free diet. Videos created by unverified individuals had the lowest quality. In contrast, unverified team accounts showed higher video quality than unverified individual ones. Additionally, verified accounts produced higher-quality videos than unverified ones, and no significant difference was observed in video quality between individual and team verified accounts.

**Table 3:** Quality assessment of Hashimoto’s disease-related-videos uploaded by different types of authors.

Author types	Verified team (*N* = 17)	Unverified team (*N* = 116)	Verified individual (*N* = 312)	Unverified individual (*N* = 155)	*p*-value
PEMAT	75 [71 ~ 80]_a,b_	75 [69 ~ 77]_a_	77 [73 ~ 85]_b_	73 [64 ~ 77]_a_	**<0.001**
VIQI	12 [11.5 ~ 14]_a_	11 [10 ~ 11]_b,c_	11 [10 ~ 12]_b_	10 [9 ~ 12]_c_	**<0.001**
GQS	3 [3 ~ 4]_a,b_	3 [3 ~ 3]_a_	4 [3 ~ 4]_b_	3 [3 ~ 3]_a_	**<0.001**
mDISCERN	3 [3 ~ 3]_a,b_	3 [3 ~ 3]_a_	3 [3 ~ 3]_b_	3 [3 ~ 3]_a_	**<0.001**
JAMA	2 [2 ~ 2]_a_	2 [1 ~ 2]_b_	2 [2 ~ 2]_a_	2 [1 ~ 2]_c_	**<0.001**

Data were presented as Median [P25 ~ P75]. Bold text means the *p*-value<0.05.

*p*-values were obtained from the Kruskal-Wallis. The Dunn Test was used for multiple comparisons: Each subscript letter denotes a subset of “Author types” categories whose column proportions do not differ significantly from each other at the 0.05 level.

Pink indicates groups that are significantly higher than at least one other group (*p* < 0.05, Dunn test), and yellow indicates groups that are significantly lower than at least one other group (*p* < 0.05, Dunn test). If two cells share the same color, it means that both groups differ significantly from at least one other group, but they are not significantly different from each other.

## Discussion

The use of social media for public health education has significantly increased, with digital video helping to overcome traditional barriers to information access (1). This study examines three video-sharing platforms: YouTube, Bilibili, and TikTok, chosen for their market dominance. YouTube, established in 2005, is the globally leading long-form video platform but remains inaccessible in China (19), where Bilibili, founded in 2009 and often referred to as “Chinese YouTube,” is the dominant platform (20). TikTok, launched by ByteDance in 2016, has experienced unprecedented growth in short-form video content and holds the record for the fastest development in digital history (21).

Several studies have shown that although social media videos enable easy access to health information, the reliability and scientific accuracy of such content are often questionable, which may contribute to the dissemination of misinformation (22, 23).

To date, no studies have comprehensively evaluated the quality of HT-related videos across major video platforms. This study features broader coverage and larger sample size, including 600 videos from three major video platforms. To minimize subjective bias, five evaluation tools were utilized. Furthermore, this study employs a novel uploader classification method, emphasizing the advantages and necessity of verified accounts in information dissemination. It also offers actionable recommendations for the public seeking reliable health information, video creators, and platform operators.

### Video characteristics

Regarding video characteristics, YouTube’s global reach and multilingual support contribute to its higher number of views and likes than that of Bilibili. However, the unique “bullet-comment” and “coin” systems of Bilibili may have promoted audience engagement, which can be particularly reflected in the ratio of comments to views. This is also a valuable practice that other platforms can learn from. Meanwhile, short videos are better suited to leveraging people’s fragmented time, which has driven the rapid rise of short video platforms exemplified by TikTok. The dissemination capabilities of TikTok have been shown to exceed those of long-video platforms that were established earlier. As demonstrated in our study, the number of likes and comments on TikTok far exceeded those on YouTube and Bilibili. This disparity in traffic among the platforms also explains the difference in the number of subscribers for uploaders on different platforms.

### Uploader characteristics

In prior studies, the classification of uploaders was subjective and obscured (6, 17, 24). For example, a speaker claimed to be a doctor in a video; however, as the account has not been verified by the platform, the authenticity of this claim remains uncertain. Nonetheless, some studies might classify it as a professional account, a classification we argue lacks rigor. Consequently, we adopted a more objective approach to classifying the uploader types based on certification status and whether the account was personal or team-based. We referred to Liu et al.’s research which provided a detailed method for identifying verified accounts (16). The variations in uploader types across different platforms partially mirror the policy directions each platform adopts for medical content creators. As a global video platform, YouTube has attracted a significant number of team-based accounts. Additionally, the platform tends to prioritize traffic allocation to these accounts, enhancing the visibility of their content in search results. However, YouTube’s certification process is notably stringent, particularly for individual medical professionals. This may be attributed to the challenges faced by the platform in verifying the credentials of overseas doctors, leading to a considerable number of personal accounts that self-identify as doctors but lack official certification. In contrast, the certification process on Bilibili and TikTok is relatively simplified. Besides, TikTok stipulates that all doctor accounts must complete the certification and their qualifications need to reach the level of attending physician or above, while Bilibili has more lenient restrictions on the identities of creators.

### Video content

Many videos have mentioned that although HT cannot be completely prevented, a good diet and regular sleep can to some extent reduce the probability of getting the disease and delay its progression. Low iodine diet, regular re-examination, drug therapy, and surgical treatment are the main therapeutic approaches for HT.

The differences in the types of uploaders also directly affect the content of the videos. The video styles of solo narration and the recording of medical consultation scenes are easy for doctors to produce, short in length, and spread quickly. This explains the constitution of video styles on TikTok. However, the higher proportion of team accounts on YouTube allows for more diverse production styles, such as TV shows and documentaries et al., which demand detailed planning and editing. The form of long videos has also expanded the variety of content on Bilibili. Additionally, under the condition of respecting the patients’ privacy and obtaining their consent, it is permitted to film real medical scenes in China. Therefore, there are many videos featuring medical scenarios on Bilibili and TikTok. Furthermore, the treatment plans for HT vary greatly depending on the patient’s condition, making it difficult to fully elaborate on them in short videos. As a result, related topics are less discussed on TikTok, while they are more common on long-video platforms.

### Video quality

Tools are essential for video assessment. All the tools in this research have been widely applied in evaluating the quality of health-related videos. The PEMAT tool, developed in 2014 by Shoemaker, with 17 questions focusing on understandability and actionability (25). Given that long-video platforms hosted some videos aimed at medical professionals, which were less comprehensible and actionable for the general public, short-video platforms achieved higher PEMAT scores. The GQS tool, introduced by Bernard in 2007, is simpler and widely used, assessing both video quality and audience engagement (26). However, its simplicity may lead to a subjective bias (16). Nagpal’s VIQI tool, developed in 2015, stresses the video quality through each image, animation, interview, video captions, and summary (27). Thus, YouTube with more video styles and team creators had the highest VIQI scores. The mDISCERN tool was refined by Singh in 2012 to assess the video materials (28), focusing on clearness, reliability, impartiality, reference, and uncertainty. Regrettably, references and areas of uncertainty were not normally mentioned in videos across nearly all platforms, which resulted in the videos across all platforms generally underperforming in these two sub-items. In 1997, Silberg et al. proposed principles for evaluating online medical information quality in JAMA (29). This tool emphasizes authorship with relevant credentials, references, currency, and conflicts of interest. Therefore, TikTok and Bilibili with relatively higher rates of verified accounts, demonstrated a clear advantage in their scores on JAMA. However, in other disease fields, the video quality scores may vary on different platforms. YouTube videos on laryngeal cancer (16) and probiotics (30) generally scored higher than those on Bilibili, contrasting with Wang’s findings on gastric cancer (18).

We found that videos produced by verified content creators, predominantly medical professionals, were generally of higher quality. This finding is in accordance with the conclusions of several previous studies, which have demonstrated that the majority of misleading information is disseminated by non-professionals (16, 17, 31). Therefore, the following recommendations are thus proposed: For the general public, when seeking HT-related information on social media, one should prioritize viewing videos posted by verified accounts. For video creators, qualified professionals are encouraged to promptly apply for account verification. For the platforms, it is essential to establish reasonable verification policies, actively guide creators toward verification, and provide more traffic support to verified accounts.

Additionally, considering some misinformation about HT, we recommend that professionals to produce some videos to debunk the rumors. As for diet, patients with HT are generally recommended to adopt a low-iodine diet, as excessive iodine intake may exacerbate the autoimmune response and lead to thyroid cell damage, but not without-iodine diet. However, patients can normally consume iodized salt to ensure the iodine requirements of the normal human body. The relationship between food allergies and Hashimoto’s thyroiditis has not been fully proved at present (32). In recent years, interest in the gluten-free diet has increased due to its potential extraintestinal anti-inflammatory effects. Consequently, many patients with Hashimoto’s thyroiditis (HT) begin this diet on their own. However, there is not yet enough evidence to recommend this dietary approach to all patients diagnosed with HT (33).

### Limitations

This study has several limitations. First, subjective evaluation may still exist, despite using five analytical tools and three trained medical professionals. Second, platform restrictions limited access to certain engagement metrics (e.g., collections/shares on YouTube, views on TikTok, and “thumbs-down” across platforms). Third, all the platform lacks advanced search functions, which may cause some videos to be overlooked. Fourth, focusing only on English and Chinese content raises concerns about cross-cultural applicability. Finally, as a cross-sectional analysis of evolving social media ecosystems, these findings require longitudinal validation. Dynamic updates to platform algorithms could also lead to non-reproducible results.

## Conclusion

Social media platforms such as YouTube, Bilibili, and TikTok can help disseminate knowledge about HT to some extent. However, the overall video quality on these platforms requires improvement. The public should prioritize viewing videos from verified accounts. Qualified professionals are encouraged to apply for verification promptly and produce high-quality HT-related videos. Reasonable certification and traffic-support policies can help deliver more high-quality videos to the audience.

## Data Availability

The raw data supporting the conclusions of this article will be made available by the authors, without undue reservation.
