# Supplementary material for: Videos on YouTube, Bilibili, TikTok as sources of medical information on Hashimoto’s thyroiditis
Source: Front Public Health. 2025 Oct 15;13:1611087. doi: 10.3389/fpubh.2025.1611087 (PMC12568602; doi:10.3389/fpubh.2025.1611087)
Supplement: Supplementary file 1 [file Data_Sheet_1.docx]

**Supplementary File 1**

**Details in Methodology**

1. **Inclusion and Exclusion criteria**

Before searching, to minimize algorithmic bias, we cleared the browser history and used a newly registered account. We visited Bilibili and Douyin(also known as the Chinese version of TikTok) by using an IP in China, whlie visiting YouTube via VPN from the international version (because YouTube is not available in China without VPN).

**1.1 Inclusion criteria**

(1) Searching Date: December 1, 2024.

(2) Searching keywords: YouTube “Hashimoto’s thyroiditis” and “Hashimoto’s disease” in English, “桥本甲状腺炎” and “桥本病” in Chinese; Bilibili and TikTok “桥本甲状腺炎” and “桥本病” in Chinese.

(3) Order: the default order.

(4) Published over a week. (According to the services from the platforms, the data like views and likes are not stable during the first week and can not accurately reflect audience engagement.)

Following the implementation of the inclusion criteria on 1 December 2024, we recorded each video’s website and rapidly-evolving metrics, including views, likes, coins, collections, shares, and the author’s followers. Due to temporal constraints, it was not possible to review the entire content of the videos and determine their exclusion status. Consequently, this step was deferred to the following day.

**1.2 Exclusion criteria**

(1) For similar or **duplicated** videos, retain the one uploaded by a certified account. If neither video is from a certified account, retain the one uploaded first.

(2) Videos that do not address topics such as anatomy, etiology, prevention, pathology, epidemiology, symptoms, examinations, diagnosis, treatment, or prognosis are considered **irrelevant** and should be excluded.

(3) Videos that are **purely advertisements** and do not provide useful medical information should be excluded. However, videos that contain informative content but also receive sponsorship should not be excluded.

(4) Videos that are in **other languages** except for English and Chinese should be excluded. While certain platforms offer automated translation and subtitle creation functionalities, concerns regarding the precision of translation remain. Therefore, we excluded videos in languages other than English and Chinese. However, if the video creator has manually added subtitles that meet the language requirements, this should be considered part of the creator’s effort and incorporated into the evaluation of the video content, and should not be excluded.

This phase of the study was conducted from December 2 to December 10, 2024. Following the application of the inclusion and exclusion criteria, the top 200 videos from each platform’s search results (600 videos in total) were retained for further analysis.

1. **Definition of author’s verification and types of video style**

We drew upon Liu’s research methods (doi: 10.1186/s12889-024-19077-6), where detailed definitions of these indicators have been provided. To minimize excessive textual overlap with Liu’s work, we have chosen not to elaborate with detailed examples.

1. **Types of video topics**
2. Anatomy
3. Etiology/Prevention: Since prevention is often discussed in conjunction with the causes, we have integrated these two topics into a single section.
4. Pathology
5. Epidemiology
6. Symptoms
7. Diagnosis
8. Treatment/Prognosis: Since prognosis is often discussed after the treatments, we have integrated these two topics into a single section.

Record the topics discussed in a video. If a video covers≥2 topics, the one that occupies the greatest proportion of the video’s duration is designated as the **main topic.**

1. **Explanation of multiple comparisons**

We take **Table 3** as an example.


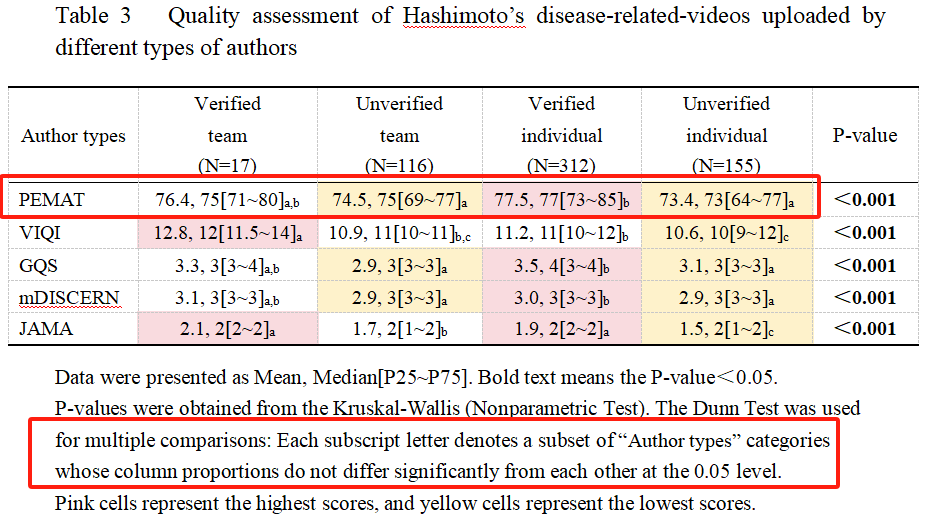


Line-PEMAT: The footnote of the verified team (VT) is “a,b”. The footnote of the unverified team (UT) is “a”. The footnote of the verified individual (VI) is “b”. The footnote of the unverified individual (UI) is “a”.

VT_a,b_ vs UT_a_: The footnotes of both groups include the letter “a”, so there is no statistically significant difference between the two groups (P＞0.05).

VT_a,b_ vs VI_b_: The footnotes of both groups include the letter “b”, so there is no statistically significant difference between the two groups (P＞0.05).

VT_a,b_ vs UI_a_: The footnotes of both groups include the letter “a”, so there is no statistically significant difference between the two groups (P＞0.05).

UT_a_ vs VI_b_: They have different footnotes, so there is a statistically significant difference between the two groups (P＜0.05).

UT_a_ vs UI_a_: They have the same footnotes, so there is no statistically significant difference between the two groups (P＞0.05).

VI_b_ vs UI_a_: They have different footnotes, so there is a statistically significant difference between the two groups (P＜0.05).
