# Supplementary material for: Videos on YouTube, Bilibili, TikTok as sources of medical information on Hashimoto’s thyroiditis
Source: Front Public Health. 2025 Oct 15;13:1611087. doi: 10.3389/fpubh.2025.1611087 (PMC12568602; doi:10.3389/fpubh.2025.1611087)
Supplement: Supplementary file 2 [file Data_Sheet_2.docx]

**Supplementary File 2**

**Details in Assessment Tools**

In order to evaluate video quality more comprehensively, we used five commonly used rating tools. Previous studies have validated the tools, particularly in the context of social media platforms. Here are the details:

1. **PEMAT** (Patient education materials assessment tool)

PEMAT is the most comprehensive tool among the tools in our study, as it consists of 25 questions, with 21 representing the understandability of health information and 4 evaluating the actionability of recommendations by videos. See Supplementary File 3 for detailed scoring criteria.

PEMAT can not shown in a Word file, see more details in Supplementary File 3 (Excel) or visit <http://www.ahrq.gov/pemat>

1. **VIQI** (Video Information and Quality Index)

The VIQI tool encompasses four dimensions including flow, accuracy, quality and precision. The score of each question ranges from 1 to 5. The total score is 5 to 20.

1. VIQI 1: information flow

The VIQI 1 in our study is according to thumbs-up, because the data of thumbs-up are available from all the three platforms.

1 ＜10 thumbs-up

2 ＜100 thumbs-up

3 ＜1000 thumbs-up

4 ＜10000 thumbs-up

5 ≥10000 thumbs-up

If a video turns off the buttom of thumbs-up (very few would do this), we rate its flow by views.

1 ＜100 views

2 ＜1000 views

3 ＜10000 views

4 ＜100000 views

5 ≥100000 views

1. VIQI 2: information accuracy
2. VIQI 3: quality (videos including one point for each image, animation, interview, video captions, and summary)
3. VIQI 4: precision (level of coherence between video title and content)

1. **mDISCERN** (Modified DISCERN)

The mDISCERN was adapted from the DISCERN tool and is more suitable for assessing video material, consist of five parts including clear aims, reliability, balance, additional reference and areas of uncertainty. The score of each question ranges from 0 to 1. The total score is 0 to 5.

1. mDISCERN-1 Are the aims clear and achieved?

0-no 1-yes

(2) mDISCERN-2 Are reliable sources of information used?

0-no 1-yes

(3) mDISCERN-3 Is the information presented balanced and unbiased?

0-no 1-yes

(4) mDISCERN-4 Are additional sources of information listed for patient reference?

0-no 1-yes

(5) mDISCERN-5 Are areas of uncertainty mentioned?

1. no 1-yes
2. **GQS (**Global Quality Score)

GQS, a 5-point scale, assesses the overall video quality, ranging from poor (1) to excellent (5). It is the simplest of all tools in our study.

(Score 1) Poor quality, poor flow of the site, most information missing, not at all useful for patients.

(Score 2) Generally poor quality and poor flow, some information listed but many important topics missing.of very limited use to patients.

(Score 3) Moderate quality, suboptimal flow, some important information is adequately discussed but others poorly discussed, somewhat useful for patients.

(Score 4) Good quality and generally good flow, most of the relevant information is listed.but some topics not covered, useful for patients.

(Score 5) Excellent quality and excellent flow, very useful for patients.

1. **JAMA** (Journal of the American Medical Association benchmark criteria)

The score of each question ranges from 0 to 1. The total score is 0 to 4.

1. Authorship: Clearly states authors/creators (individuals or organizations) with relevant credentials.

Example: “Produced by a board-certified cardiologist.”

1. Attribution: Sources of information are listed (e.g., peer-reviewed studies, clinical guidelines).

Example: “References to NIH guidelines are hyperlinked.”

1. Currency: Provides dates of creation/update to ensure timeliness.

Example: “This video was reviewed in March 2024.”

1. Transparency: Discloses conflicts of interest (e.g., sponsorships, funding sources).

Example: “This video is funded by an educational grant from [non-profit organization].”

- Consistency analysis

To avoid biases, two evaluators independently assessed and rated video quality using validated measurement tools. Discrepancies in ratings were adjudicated through consultation with a third researcher to establish consensus. The consistency between the two raters were analyzed.

Since this study employed five different scales, and each scale had several sub-items. To simplify the calculation process, we only calculated the total scores of each scale. Note: PEMAT separately calculates the total score for understandability (PEMAT-U) and the total score for actionability (PEMAT-A). For PEMAT and VIQI (continuous variables), we used the intraclass correlation coefficient (ICC). For GQS, mDISCERN, and JAMA (ordered categorical variable), we used weighted kappa.

Results:

PEMAT-U, ICC=0.962.

PEMAT-A, ICC=0.937.

VIQI, ICC=0.908.

GQS, weighed Kappa=0.832

mDISCERN, weighed Kappa=0.802

JAMA, weighed Kappa=0.922
